# On the Need to Determine the Contribution of Anti-Nucleocapsid Antibodies as Potential Contributors to COVID-19 Convalescent Plasma Efficacy

**DOI:** 10.3390/v14112378

**Published:** 2022-10-27

**Authors:** Daniele Focosi, Massimo Franchini, Arturo Casadevall

**Affiliations:** 1 North-Western Tuscany Blood Bank, Pisa University Hospital, 56124 Pisa, Italy; 2 Division of Hematology and Transfusion Medicine, Carlo Poma Hospital, 46100 Mantua, Italy; 3 Department of Molecular Microbiology and Immunology, Johns Hopkins Bloomberg School of Public Health, Baltimore, MD 21287, USA

**Keywords:** COVID-19, SARS-CoV-2, nucleocapsid, protein localization, antibody therapy

## Abstract

Historically the therapeutic potential of polyclonal passive immunotherapies in viral diseases has been related to antiviral neutralizing antibodies, but there is also considerable evidence that non-neutralizing antibodies can translate into clinical benefit as well. In the setting of SARS-CoV-2 infection, we review here in vitro and in vivo evidence supporting a contributing role for anti-nucleocapsid antibodies. Retrospective investigation of anti-nucleocapsid antibody levels in randomized clinical trials of COVID-19 convalescent plasma is warranted to better understand whether there is an association with efficacy or lack thereof.

Many patients with infectious diseases cannot tolerate the side effect of small chemical antimicrobials, making antibody a safer prophylactic and therapeutic alternative. This is case for immunocompromised individuals with COVID-19 where the ritonavir component of the Paxlovid antiviral formulation can interfere with other drugs needed to treat their underlying condition. When matching viruses and therapeutic antibodies, most attention has focused so far on neutralizing antibodies (nAb), i.e., antibodies of different isotypes (IgG_1_, IgG_3_, IgM, and IgA) that reduce viral infection of replication-competent cells in in vitro viral neutralization tests (VNT) [1]. With regards to SARS-CoV-2, the causative agent of COVID-19, this in vitro neutralization reflects largely the presence of antibodies to the region of the Spike protein that interacts with the ACE2 receptor on human cells. Hence, for COVID-19, nAbs consist mostly of antibodies to the Spike protein. B-cells making these antibodies recovered from convalescent donors have been used to make the therapeutic monoclonal antibodies. Furthermore, the titer of nAbs has been the major correlate of protection after Spike-based vaccination used to assess vaccine efficacy [2]. For COVID19 convalescent plasma (CCP), the therapeutic potential has been generally correlated with the nAb titer. This assumption has been validated on both mechanistic studies that established its antiviral activity [3], and clinical studies that show dose-response relationships between the nAb titer and efficacy [4,5,6]. However, the undoubted importance of nAbs does not exclude the possibility that more antiviral antibodies could be associated with additional clinical benefits. This principle was illustrated in studies with vesicular stomatitis virus where in vitro neutralization titer correlated with avidity and neutralization rate constant, but in vivo efficacy was independent of in vitro neutralizing activity (PMID: 9197261).

In contrast to the SARS-CoV-2 Spike protein, the structural nucleocapsid (N) phosphoprotein is highly conserved among human coronaviruses, where it is essential for linking the viral genome to the viral membrane [7]. N is classically considered an internal protein of SARS-CoV-2, and, as such, to be only useful for eliciting T-cell mediated immune responses [8,9]. Orthologous N protein from influenza A [10], measles [11], respiratory syncytial [12], lymphocytic choriomeningitis [13], and human immunodeficiency viruses [14] are expressed on the surface of infected cells, where they can be the target for antibody dependent cellular cytotoxicity mechanism (ADCC). Not surprisingly, about 10^4^–10^5^ SARS-CoV-2 N proteins occur on the surface of a range of different SARS-CoV-2-infected cell types, either natural (Vero, Caco-2, Calu-3) or humanized (BHK-21_hACE2 or CHOK1_hACE2, and HEK293-FT_hACE2) [15]. SARS-CoV-2 N is likely placed on the cell surface and secreted through a non-canonical pathway that bypasses insertion into the endoplasmic reticulum [16]. N released by SARS-CoV-2 infected cells or N-expressing transfected cells binds to heparan sulfate, which promotes Spike-ACE2 interaction [17] and heparin on neighboring cells, which may contribute to coagulopathy, and also neutralizes the biological activity of many different chemokines, blocking chemotaxis of immune effector cells [15]. Freely circulating N protein can also activate the complement cascade via the alternative pathway, thus potentially contributing to the inflammatory changes that are associated with severe COVID-19 [18,19]. Hence, there is considerable direct and circumstantial evidence that the N protein contributes to the pathogenesis of COVID-19, and if that is the case, it is reasonable to posit that N-binding antibodies can contribute to host defense through ADCC, and by interfering with its deleterious effects on immune function.

Antibodies to the N protein are elicited after immunization with experimental N-based vaccines [8,9,20,21] and after natural SARS-CoV-2 infection. In the pre-vaccine era, the occurrence of antibodies to N following natural infection was almost universal and levels remained detectable for more than 6 months [22,23,24]. The finding that each 1-log increase in SARS-CoV-2 viral copies at diagnosis was associated with 90% higher odds of seroconversion for N antibodies [25] suggests that high levels of circulating N, which are also associated with the severity of pulmonary illness and clinically important patient outcomes [26], are required to elicit this response.

Whether antibodies to the N protein exert any protective role has been the subject of sporadic investigations. mAbs targeting SARS-CoV-2 N protein can inhibit free N-induced MASP-2 activation in vitro [18], and mAbs to the related alphacoronavirus mouse hepatitis virus (MHV) N protein exert anti-viral activity in vitro in the presence of complement and in vivo [27,28]. A very interesting animal model showed that C57BL/6 mice prime-boosted with an adenovirus serotype five vector expressing N developed anti-N antibodies 2 weeks later, which were unable to neutralize live authentic SARS-CoV-2. However, when their sera was transfused to *naïve* K18-hACE2 mice, followed by intranasal challenge with 10^3^ PFU SARS-CoV-2 USA-WA1/2020, the animals experienced lung viral loads 14-fold lower than in controls at day 4 [29]. Very few clinical studies have investigated the correlation between antibody levels to N protein in CCP and clinical outcome. In a retrospective observational study in 96 hospitalized patients, Cain et al. found no statistically significant difference in neither mortality nor time from transfusion to death between patients receiving CCP with low vs. high antibody levels to N protein. Unfortunately no multivariate analysis was conducted to account for antibody levels to spike protein in the plasma [30]. When it comes to randomized controlled trials (RCT), the gold standard of evidence-based medicine, only a single RCT assessed antibody to N protein in CCP. A reanalysis of the Penn2CCP RCT data showed that the clinical benefit of CCP was related to a shift towards reduced inflammatory S responses and enhanced N humoral responses [31]. Furthermore, CCP induced immunomodulatory changes to recipient humoral profiles (including more anti-inflammatory S-specific Fc glycans) persisted for at least 2 months, marked by the selective evolution of anti-inflammatory Fc-glycan profiles and persistently expanded N-specific humoral immunity following CCP therapy [32].

Many different ingredients in CCP can lead to clinical benefit [33] and there is now suggestive evidence for adding antibodies to N protein to the list of potential antiviral ingredients. Mechanisms of action other than neutralization are needed to explain the potential clinical benefit in vivo of antibodies to N protein. In this regard, antibodies to the SARS-CoV-2 N protein, once bound to the surface of N-expressing cells, activate Fc receptors (FcR)-expressing cells [15]. In murine influenzavirus models, IgG to N protein specifically promoted virus clearance by using a mechanism involving both FcRs and CD8^+^ T lymphocytes [34]. Accordingly, antibodies to SARS-CoV-2 N protein exert relevant antibody-dependent NK cell activation (ADNKA) after infection, driving high levels of pro-inflammatory cytokine production for more than 6 months [35].

Of interest and in contrast to the highly variable Spike protein, N protein has been mostly conserved so far in SARS-CoV-2 evolution, with antibodies to N showing cross-reactivity across sublineages [36]. Hence, if any, the therapeutic benefit from old CCP units could be preserved against the most recent SARS-CoV-2 variants of concern. However, there is great cross-reactivity for antibodies to N proteins among coronaviruses, and N protein epitopes are shared between SARS-CoV-2 and alphacoronaviruses. Hence, one cannot be certain whether any correlation between antibody to N protein and protection was a result from previous endemic coronavirus infection(s), which are highly prevalent in the human populations worldwide, or from the recent SARS-CoV-2 infection. Nevertheless, the amino acid sequences of the entire N protein of common coronaviruses are sufficiently dissimilar to that of SARS-CoV-2, with only the conserved residues at the N-terminal domain of NP showing a high degree of similarity. Consequently, usage of an N-terminally truncated nucleocapsid protein (ΔN-NP) could provide better specificity for discriminating among coronaviruses [37], with epitope mapping unveiling the 155–171 epitope [38] and 255–346 [39] as highly immunogenic and specific. High titer responses against N of alphacoronaviruses have been detected during early COVID-19 stages, raising the possibility that SARS-CoV-2 infection boosted pre-existing immunity [40], without clear correlations with disease severity [41]. That said, it is likely that not all anti-N antibodies are equally beneficial: e.g., occurrence of antibodies to a 21-residue epitope from N (termed Ep9) [42], lack of antibodies against the seasonal betacoronavirus OC43 N [43], or occurrence of IgG to -alphacoronaviruses (NL-63 and 229E) N protein [40] have all been associated with severe COVID-19. Notably, in individuals with severe COVID-19 (such as those admitted to ICU), N-specific antibody titers prevail over anti-Spike titer [40,44,45].

There is unfortunately an unpredictable conundrum between the increased potency of CCP from vaccinated donors (so-called VaxPlasma or “hybrid plasma” or VaxCCP) and anti-N antibodies. The superiority of VaxCCP over CCP is due to the 10–100-fold higher anti-Spike nAb titers seen in VaxCCP compared to CCP, and their heterologous nature neutralizing most, if not all, SARS-CoV-2 variants of concern. Nevertheless, it has been shown that after vaccine breakthrough infection the occurrence of antibody to N protein, compared to unvaccinated subjects, is dramatically decreased from 93% to just 50% at day 54 post-infection [22,25]. The few individuals that mount antibody responses to N protein after vaccine breakthrough infection make lower titers [46], and substantial seroreversion of N total immunoglobulin has also been found shortly after vaccine breakthrough infections [47]. As such, VaxCCP has much lower content of anti-N, which will be an unlikely contributor to clinical benefit in the future usages.

The available evidence provides a compelling case for analyzing the antibody content to N protein on stored samples from the dozens of RCTs completed studying CCP efficacy against COVID-19. There are thousands of such CCP remnant samples available that could be studied for antibodies to N and correlated to clinical outcome data. Although the results of such a study will likely have no immediate implications for the current pandemic, it has the potential to inform future pandemics from related coronaviruses or different viruses expressing orthologous protein N’s. At the very least, we should miss this opportunity to further dissect the contribution of humoral immunity to N protein in COVID-19. In particular, under the upcoming “mutations wave” dominated by Spike R346X- and K444X-harboring Omicron sublineages, CCP will remain a fundamental weapon against COVID19 in immunocompromised patients [48], who are at higher risk for more severe presentation and are not protected by vaccine boosts.

## Data Availability

This manuscript generated no new data.
